# Consequences of LED Lights on Root Morphological Traits and Compounds Accumulation in *Sarcandra glabra* Seedlings

**DOI:** 10.3390/ijms22137179

**Published:** 2021-07-02

**Authors:** Dejin Xie, Muhammad Waqqas Khan Tarin, Lingyan Chen, Ke Ren, Deming Yang, Chengcheng Zhou, Jiayi Wan, Tianyou He, Jundong Rong, Yushan Zheng

**Affiliations:** 1 College of Forestry, Fujian Agriculture and Forestry University, Fuzhou 350002, China; 2160482002@fafu.edu.cn (D.X.); 1190428010@fafu.edu.cn (K.R.); 2180428006@fafu.edu.cn (D.Y.); 3190422030@fafu.edu.cn (J.W.); rongjd@fafu.edu.cn (J.R.); 2 College of Landscape Architecture, Fujian Agriculture and Forestry University, Fuzhou 350002, China; waqas_tarin@yahoo.com (M.W.K.T.); fafucly@fafu.edu.cn (L.C.); 1181775051@fafu.edu.cn (C.Z.); hetianyou@fafu.edu.cn (T.H.)

**Keywords:** *Sarcandra glabra*, LED light, root tissue, transcriptomic, metabolomic, phenylpropanoid-derived, terpenoids

## Abstract

This study evaluated the effects of different light spectra (white light; WL, blue light; BL and red light; RL) on the root morphological traits and metabolites accumulation and biosynthesis in *Sarcandra glabra*. We performed transcriptomic and metabolomic profiling by RNA-seq and ultra-performance liquid chromatography−electrospray ionization−tandem mass spectrometry (UPLC-ESI-MS/MS), respectively. When morphological features were compared to WL, BL substantially increased under-ground fresh weight, root length, root surface area, and root volume, while RL inhibited these indices. A total of 433 metabolites were identified, of which 40, 18, and 68 compounds differentially accumulated in roots under WL (WG) vs. roots under BL (BG), WG vs. roots under RL (RG), and RG vs. BG, respectively. In addition, the contents of sinapyl alcohol, sinapic acid, fraxetin, and 6-methylcoumarin decreased significantly in BG and RG. In contrast, chlorogenic acid, rosmarinyl glucoside, quercitrin and quercetin were increased considerably in BG. Furthermore, the contents of eight terpenoids compounds significantly reduced in BG. Following transcriptomic profiling, several key genes related to biosynthesis of phenylpropanoid-derived and terpenoids metabolites were differentially expressed, such as caffeic acid 3-*O*-methyltransferase) (*COMT*), hydroxycinnamoyl-CoA shikimate hydroxycinnamoyl transferase (*HCT*), O-methyltransferase (*OMT*), and 1-deoxy-D-xylulose-5-phosphate synthetase (*DXS*). In summary, our findings showed that BL was suitable for growth and accumulation of bioactive metabolites in root tissue of *S. glabra*. Exposure to a higher ratio of BL might have the potential to improve the production and quality of *S. glabra* seedlings, but this needs to be confirmed further.

## 1. Introduction

*Sarcandra glabra* (Thunb.) Nakai is an evergreen Chinese herb distributed in southern China. When dry, the whole plant is used as a traditional medicine for the treatment of inflammation [1], bacterial [2], oxidant stress [3], and tumor [4] (The state Pharmacopoeia Commission of People’s Republic of China). Until now, research on *S. glabra* has concentrated on isolation and identification of various compounds in the herb, such as isofraxidin, scopoletin, and rosmarinic acid [5,6,7]. However, only a few studies have explored *S. glabra* seedling’s cultivation and growth, functional genes analysis, and metabolite biosynthesis.

In recent years, Good Agricultural Practice (GAP) project has ensured the primary environmental and operational conditions for safe, wholesome crops and herbs, and has been extensively promoted in some species, such as *Panax notoginseng* [8] and *Dendrobium candidum* [9]. The energy sustainable greenhouse cultivation is regarded as an intensive production system where light is an essential component for photosynthesis. Light-emitting diodes (LED) are an alternative and efficient light resource, which is more beneficial to plant’s growth and development [10]. In previous studies, LED lights triggered a cascade of responses in plant morphology, physiology, and metabolites accumulation in herbs (medicinal plants) and crops [11,12,13,14]. For instance, when exposed to a high percentage of BL, the hypocotyl length and leaf area of tomato and cucumber seedlings were suppressed [13,15]. Under RL condition, the callus culture of *Rhodiola imbricata* accumulated larger biomass, while the callus culture accumulated maximum amount of Salidroside in BL condition [11]. In addition, the BL significantly increased the contents of flavonoids production in *Cyclocarya paliurus* [12]. In earlier research, we observed that RL considerably increased plant height and decreased stem diameter and leaf area, while BL significantly reduced plant height and leaf area of *S. glabra*. Meanwhile, the BL substantially reduced the production of esculetin, caffeic acid, isofraxidin, and fraxidin, but accumulated the contents of quercitrin and kaempferol in leaves of *S. glabra* [16]. Under RL irradiation, the contents of cryptochlorogenic acid, cinnamic acid, and kaempferol significantly decreased in leaves of *S. glabra* [16].

Apart from the effects of various light spectra on the growth of aerial parts, light is also essential for effective root formation. All plant root systems perform the functions of water and nutrient absorption as well as soil anchoring. Notably, roots are highly flexible and adapt to changing environmental conditions both developmentally and physiologically. Recent studies have demonstrated that light also modulated the processes of root growth and development in *Arabidopsis* through different light-signaling components and pathways [17,18]. Exposure of sweet basil (*Ocimum basilicum* L.) cuttings to BL shortened the time required for root formation [19]. Compared to WL, exposure of tomato seedlings to RL significantly reduced the fresh and dry root weight, while BL did not cause any difference [20]. The lower ratio of red: far-red light stimulated the adventitious roots in Chrysanthemum [21]. The highest weight of newly formed roots in transplanted pomegranate (*Punica granatum* L.) seedlings was obtained under LED light treatment (L20AP67) [22]. Furthermore, in cherry rootstock, the RL was more effective than BL on root elongation [23]. The use of dicot plants such as *Arabidopsis* and monocot species (rice or maize) roots as models are ideal experimental methods due to the complex network of root growth and development [24,25]. Several previous studies have analyzed the molecular basis of root growth and formation in more detail [24,26]. Furthermore, various studies in the *Arabidopsis* root showed that different hormones played vital roles in the regulation of the primary root, lateral root, and root hair development, including auxins [27], abscisic acid [28], brassinosteroids [29], cytokinins [30], gibberellins [31,32] and ethylene [33,34], among others [35,36].

In the current study, we compared the effects of three LED lights [white light (WL), red light (RL), and blue light (BL)] on the root tissues of *S. glabra* seedlings by transcriptomic and metabolomic profiling further. We hypothesized that distinct light spectra might influence the root growth and accumulation of secondary metabolites in *S. glabra*. In metabolomic profiling, a comparative analysis of the identified compounds was conducted to reveal the differences between WG (root tissue under WL), RG (root tissue under RL), and BG (root tissue under BL). Meanwhile, we performed transcriptomic profiling to analyze the differentially expressed genes (DEGs) among these treatments. Simultaneously, we identified the DEGs involved in phenylpropanoid and terpenoids biosynthesis, respectively. The study objectives were to study the differences in root growth and accumulation of secondary metabolites under different light spectra treatments. Our findings will provide novel insights into the influence of the different monochromatic LED lights on root growth and metabolite accumulation patterns, which will lay the foundation for breeding and optimizing the cultivation conditions of *S. glabra* seedlings in future.

## 2. Results

### 2.1. Effects of Different Light Qualities on Root Morphology

**Figure 1:**
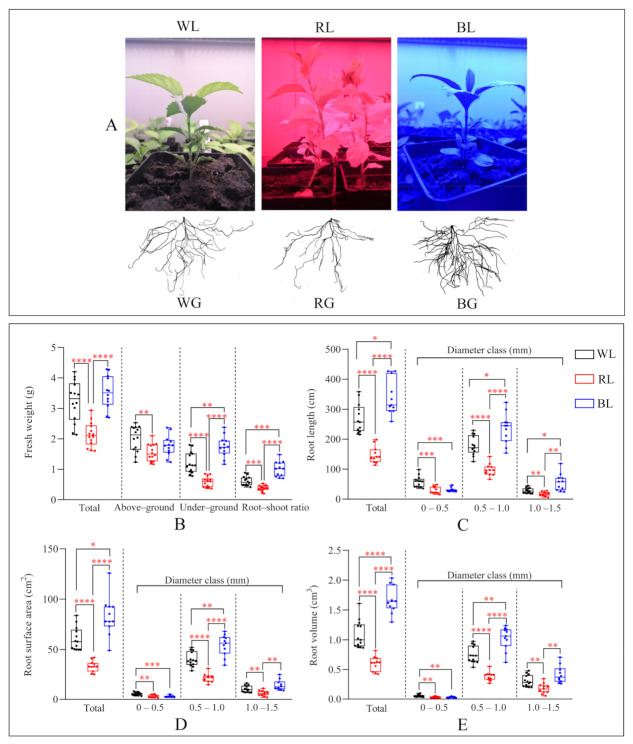
Root morphological traits under different LED lights. (**A**) *S. glabra* seedlings grown under different LED lights and the corresponding root morphology; (**B**) Fresh weight of different tissues and the root-shoot ratio; (**C**) Root length; (**D**) Root surface area; (**E**) Root volume. Maximum and minimum values are represented at the upper and lower ends of the whisker, respectively. The 75th and 25th percentiles are represented at the upper and lower ends of the box, respectively. * represents *p* < 0.05, ** represents *p* < 0.01, *** represents *p* < 0.001, **** represents *p* < 0.0001. WL: white light; RL: red light; BL: blue light; WG: root tissue under WL; BG: root tissue under BL; and RG: root tissue under RL.

After a 60-day cultivation period (Figure 1A), the fresh weight of the entire plant was significantly higher in the WL and BL treatments relative to the RL treatment. For the above-ground parts (leaves and stems), the fresh weight was significantly greater in the WL treatment in comparison with the RL and BL treatments. In contrast, the fresh weight of roots was significantly greater in the BL treatment relative to the WL and RL treatments. Consequently, the root-shoot ratio was significantly higher in the BL treatment relative to the WL and RL treatments. However, both the fresh weight of above or below ground parts and the root-shoot ratio were significantly lower in the RL treatment (Figure 1B). In addition, the total root length (Figure 1C), total root surface area (Figure 1D), and total root volume (Figure 1E) in BG were significantly higher than WG. In contrast, the values for these indices decreased significantly in RG, relative to WG. Finally, the 0.5–1.0 mm diameter class constituted the largest part of root length, root surface area and root volume, accounting for 66.6% ~ 71.0%, 64.8% ~ 66.6% and 61.6% ~ 68.7%, respectively (Supplementary Table S1).

### 2.2. Metabolites Analysis of Roots under Different LED Lights

A total of 433 metabolites were evaluated, including flavonoids (76), lipids (59), amino acids and derivatives (52), phenolic acids (64), alkaloids (39), organic acids (32), terpenoids (10), lignans and coumarins (10) among others (Supplementary Table S2).

**Figure 2:**
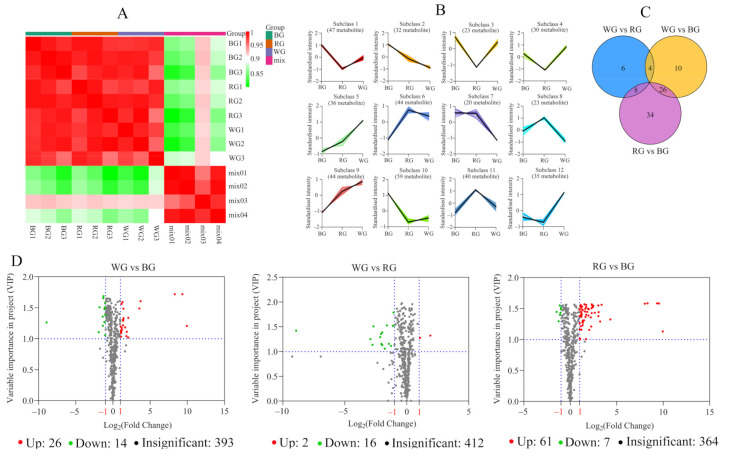
Metabolite profiling of root tissues under different LED lights. (**A**) Correlation analysis between different samples; (**B**) K-means clustering analysis in WG, BG, and RG, and the *y*-axis showing standardized intensity that the relative contents were calculated by centroid initialization and standardized processing; (**C**) Venn diagram showing the similarities and differences (**D**) Volcano map showing log_2_ (Fold change) and VIP. If VIP ≥ 1 and Fold change ≥ 2 or ≤ 0.5, they were regarded as the differential metabolites.

The results of correlation analysis (Figure 2A) showed that the biological replicates of different treatments had similar accumulation patterns of metabolites (R^2^ > 0.9). K-means clustering analysis revealed that all the detected metabolites could be divided into 12 subclasses (Figure 2B and Supplementary Table S3), with each subclass consisting of metabolites with similar accumulation patterns among three root tissues. In subclass 1, 2, 3, and 10, the contents of compounds were higher in BG; in subclass 6, 8, and 11, the production of metabolites strongly accumulated in RG. However, in subclass 4, 5, 9, and 12, metabolites had higher contents in WG. For Venn diagram (Figure 2C) and Volcano plots (Figure 2D), there were 40 metabolites that were differentially accumulated (26 up-regulated and 14 down-regulated) between WG and BG (Supplementary Table S4). In contrast, only 18 metabolites had significantly differential accumulation patterns (2 up-regulated and 16 down-regulated) between WG and RG (Supplementary Table S5). Meanwhile, there were 68 metabolites that were differentially accumulated (61 up-regulated and seven down-regulated) between RG and BG (Supplementary Table S6). The differential metabolites mainly enriched the biosynthesis of secondary metabolites and metabolic pathways. 

**Figure 3:**
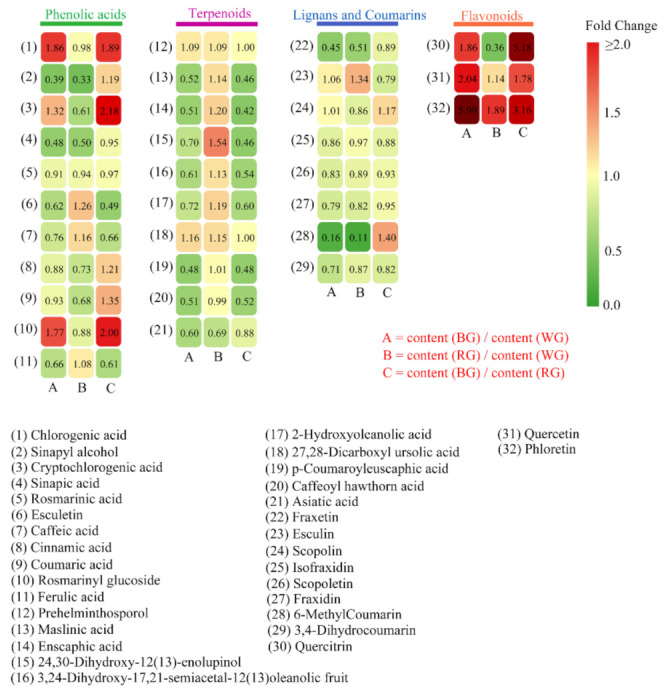
Heat map of the fold change of the relative contents of the 32 active metabolites. In the upper legend, different colors represent different metabolite classes. The metabolite names are listed at the bottom. All the fold changes in relative content values of the metabolites are shown in the right legend. A: the fold change of relative content between BG and WG; B: the fold change of relative content between RG and WG; C: the fold change of relative content between BG and RG. Fold change ≥ 2 or ≤ 0.5 were regarded as the differential metabolites.

Meanwhile, we selected several major ingredients of *S. glabra* to study the effect of different LED lights on their accumulation patterns in roots (Figure 3 and Supplementary Table S7). Among phenolic acids, sinapyl alcohol and sinapic acid decreased significantly in BG and RG, while the contents of chlorogenic acid and rosmarinyl glucoside increased substantially in BG. Meanwhile, BL inhibited the accumulation of esculetin in the root, while RL slightly stimulated it. As for lignans and coumarins, the contents of fraxetin and 6-methylcoumarin significantly reduced in BG and RG, relative to WG. The production of isofraxidin, scopoletin, fraxidin, and 3, 4-dihydrocoumarin was reduced to various degrees. Within the flavonoid class, the content of quercitrin was much higher in BG, but it decreased remarkably in RG. The production of quercetin and phloretin significantly increased in BG, and the contents of them in RG were 1.1 and 1.9-fold higher than that in WG, respectively. As for terpenoids, only nine triterpenoids and one sesquiterpenoid (prehelminthosporol) were identified in this study. Except for prehelminthosporol and 27, 28-Dicarboxyl ursolic acid, the contents of other terpenoids in BG were from 0.5- to 0.7-fold lower than that in WG. Meanwhile, the contents of 24,30-Dihydroxy-12(13)-enolupinol and asiatic acid in RG were 1.5- and 0.7-fold than that in WG, whereas other terpenoids did not show any considerable change in RG.

### 2.3. Differentially Expressed Genes among the Roots under Three LED Lights

**Figure 4:**
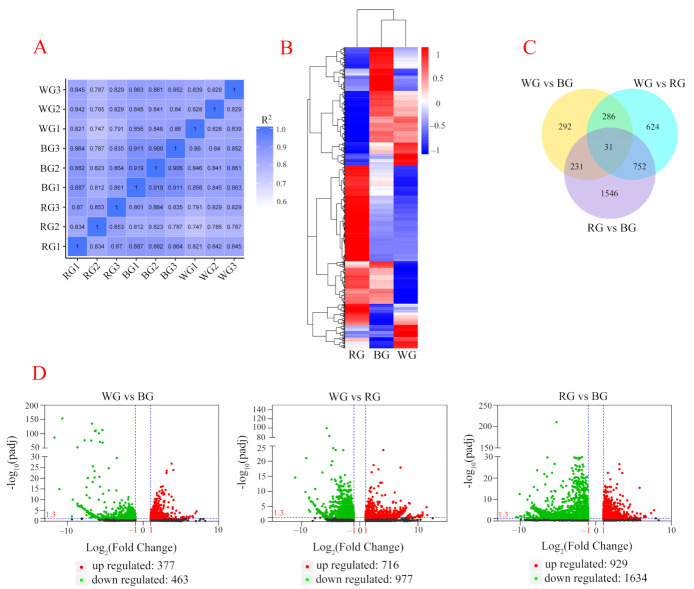
Transcriptomic profiling of root tissues under different LED lights. (**A**) correlation analysis between different samples; (**B**) Heatmap and hierarchical clustering showing DEGs in WG, BG, and RG; (**C**) Venn diagram showing the distribution of DEGs from WG, BG, and RG; (**D**) Volcano map showing the log_10_ (padj) and log_2_ (fold change) of DEGs. If −log_10_(padj) ≥ 1.3 and Fold change ≥ 2 or ≤ 0.5 were regarded as differentially expressed genes.

For transcriptomic profiling, a total of 68,086,464, 62,545,542, and 59,542,243 clean reads were obtained from WG, BG, and RG, respectively. Correlation analysis results (Figure 4A) showed that the three biological replicates in each treatment were similar (R^2^ > 0.8). The results of hierarchical clustering analysis revealed that WG and BG were classified into the same clade, while the expression profile of RG was different from that of other treatments (Figure 4B). The results of the DEG analysis showed that 3762 genes were significantly expressed (higher or lower) among WG, BG, and RG (Figure 4B). Among them, 840 (WG vs. BG), 1693 (WG vs. RG), and 2560 (RG vs. BG) DEGs had significant differences in expression profiles (Figure 4C,D, and Supplementary Table S8).

**Figure 5:**
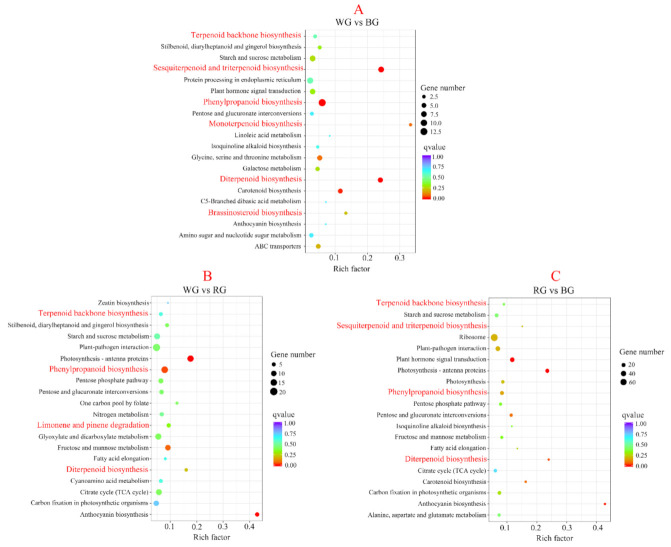
The most enriched KEGG pathways in the pairwise comparison between WG vs. BG, WG vs. RG, and RG vs. BG. (**A**) KEGG enrichment analysis in WG vs. BG group; (**B**) KEGG enrichment analysis in WG vs. RG group; (**C**) KEGG enrichment analysis in RG vs. BG group. The size and color of solid circles represent the number of DEGs involved in the specific pathway and the significant value (*q* value) of the rich factor, respectively.

Meanwhile, all DEGs were mapped in the KEGG database. We found that the DEGs significantly enriched KEGG pathways related to terpenoids metabolism and phenylpropanoid biosynthesis (Figure 5A–C). Therefore, our study mainly focused on these metabolite pathways.

### 2.4. Candidate Genes Involved in the Biosynthesis of Phenylpropanoids and Terpenoids

**Figure 6:**
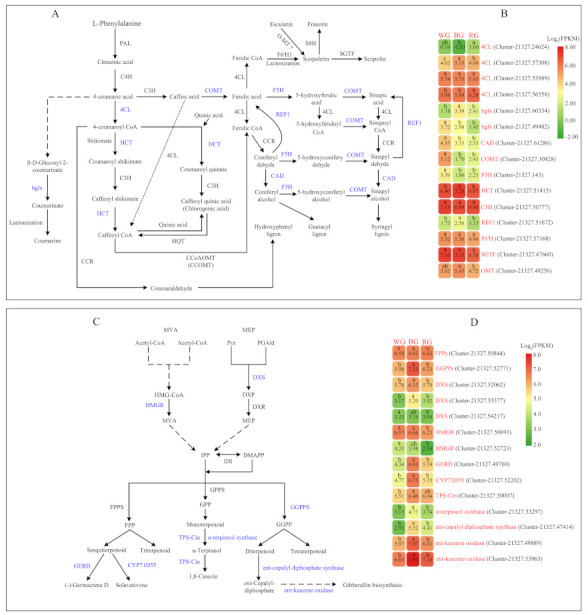
Phenylpropanoid (**A**) and terpenoids (**C**) metabolism, biosynthesis, and the heat map of the unigenes (**B**,**D**) under WG, BG, and RG in *S. glabra*. *PAL*, phenylalanine ammonia lyase; *C4H*, cinnamate 4-hydroxylase; *4CL*, 4-coumaroyl CoA ligase; *C3H*, coumarate 3-hydroxylase; *HCT*, hydroxycinnamoyl-CoA shikimate hydroxycinnamoyl transferase; *HQT*, hydroxyl-cinnamoyl CoA quinate hydroxycinnamoyl transferase; *COMT*, caffeic acid 3-*O*-methyltransferase; *CCoAOMT* (*CCOMT*), caffeoyl CoA 3-*O*-methyltransferase; F6’H1, feruloyl-CoA 6´-Hydroxylase 1; *S8H*, scopoletin 8-hydroxylase; *SGTF*, scopoletin glucosyltransferase; *OMT*, O-methyltransferase; *bglx*, beta-glucosidase; *F5H*, ferulate-5-hydroxylase; *CAD*, cinnamyl alcohol dehydrogenase; *REF1*, coniferyl-aldehyde dehydrogenase; Pyr, pyruvic acid; PGAld, glyceraldehyde-3-phosphate; MEP, 2-C-methyl-d-erythritol-4-phosphate; DXP, 1-deoxy-d-xylulose-5-phosphate; MVA, mevalonate; HMG-CoA, 3-hydroxy-3-methyl Glutaryl coenzyme A; IPP, isopentenyl diphosphate; DMAPP, dimethylallyldiphosphate; GPP, geranyl pyrophosphate; FPP, farnesyl pyrophosphate; GGPP, geranylgeranyl diphosphate; *DXS*, 1-deoxy-D-xylulose-5-phosphate synthetase; *HMGR*, 3-hydroxy-3-methyl Glutaryl-CoA reductase; *IDI*, isopentenyl diphosphate isomerase; *GPPs*, geranyl pyrophosphate synthase; *FPPs*, farnesyl pyrophosphate synthase; *GGPPs*, geranylgeranyl diphosphate synthase; *TPS-cin*, 1,8-cineole synthase; *GERD*, (-)-germacrene D synthase; *CYP71D55*, premnaspirodiene oxygenase; the names of the various unigenes are listed on the vertical line, and the samples are listed horizontally. The scale represents the logarithms of the FPKM values of these unigenes based on transcriptome data. Different lower cases indicate significant differences (*p* < 0.05) based on Duncan’s multiple range test. The enzymes encoded by corresponding DEGs are indicated in blue color. Dotted lines indicate that some steps of enzymatic reactions are omitted.

Candidate genes were selected according to the annotation of *S. glabra* transcriptome, after which their expression levels in WG, BG, and RG were analyzed (Supplementary Table S9). In the phenylpropanoid pathway (Figure 6A,B), a total of 11 genes were significantly up or down-regulated. Four *4CL* (4-coumaroyl CoA ligase) candidate unigenes were identified, of which Cluster-21327.56558 and Cluster-21327.24624 were significantly expressed in RG, while Cluster-21327.57308 was highly expressed in BG. The pathway for the production of chlorogenic acid, the unigene of Cluster-21327.51415 encoding HCT (hydroxycinnamoyl-CoA shikimate hydroxycinnamoyl transferase) enzyme was significantly up-regulated. The unigenes of Cluster-21327.60334 and Cluster-21327.49482 encoding the bglx (beta-glucosidase) enzyme were related to coumarin biosynthesis, and the former showed higher expression in BG. As for lignin biosynthesis, four candidate genes were screened, including *F5H* (Cluster-21327.143, ferulate-5-hydroxylase), *COMT* (Cluster-21327.30828, caffeic acid 3-O-methyltransferase), *CAD* (Cluster-21327.61286, cinnamyl alcohol dehydrogenase), and *REF1* (Cluster-21327.51672, coniferyl-aldehyde dehydrogenase). Compared to WG, the expression levels of *F5H*, *COMT*, and *CAD* were significantly down-regulated in BG and RG, whereas *REF1* had a higher expression level in BG. In scopolin biosynthesis, both Cluster-21327.47660 encoding SGTF (scopoletin glucosyltransferase) and Cluster-21327.49256 encoding OMT (O-methyltransferase) were expressed at a significantly lower level in RG.

The pathway for terpenoid biosynthesis (Figure 6 C,D), can be divided into several discrete processes. At the early steps, all the terpenoids are derived from MEP (2-C-methyl-d-erythritol-4-phosphate) and MVA (mevalonate) pathways, which supply the precursors IPP (isopentenyl diphosphate) and DMAPP (dimethylallyl diphosphate), respectively. DXS (1-deoxy-d-xylulose-5-phosphate synthetase) and HMGR (3-hydroxy-3-methyl Glutaryl-CoA reductase) are the rate-limiting steps in the MEP and MVA pathway, respectively. This study identified three unigenes encoding DXS enzyme and two unigenes that encode the HMGR enzyme. For *DXS* genes, Cluster-21327.52062 and Cluster-21327.55377 were highly expressed in BG, but they had lower expression levels in WG and RG. Additionally, the expression level of Cluster-21327.54217 in WG and BG had no substantial differences, while it was down-regulated in RG. Among *HMGR* candidate unigenes, the expression levels of Cluster-21327.50693 and Cluster-21327.52723 were significantly lower in RG. In the following steps, the formation of various terpenoid skeletons was initiated from GPPs (geranyl pyrophosphate synthase), FPPs (farnesyl pyrophosphate synthase), and GGPPs (geranylgeranyl diphosphate synthase) enzymatic reactions. The unigene of Cluster-21327.52771 encoding GGPPs enzyme had a higher expression level in BG, whereas Cluster-21327.50844 showed no significant differential expression in WG, BG, and RG. At the final processes, all types of terpenoids were produced by some specific enzymes. As for the biosynthesis of monoterpenoids, both Cluster-21327.50007 encoding TPS-cin (1,8-cineole synthase) and Cluster-21327.53297 encoding α-terpineol synthase were significantly up-regulated in BG, contributing to increasing higher production of 1,8-cineole. In sesquiterpenoid biosynthesis, Cluster-21327.49769 encoding GERD [(-)-germacrene D synthase] and Cluster-21327.52202 encoding CYP71D55 (premnaspirodiene oxygenase) had higher expression levels in BG, showing positive effects on the accumulation of (-)-Germacrene D and Solavetivone compounds. Furthermore, the expression level of Cluster-21327.47414 encoding ent-copalyl diphosphate synthase, Cluster-21327.49889, and Cluster-21327.53963 encoding ent-kaurene oxidase were significantly up-regulated in BG. Thus, it was likely that BL had a significant regulatory influence on diterpenoid biosynthesis in the root tissue, primarily through the gibberellin pathway.

### 2.5. qRT-PCR Verification

**Figure 7:**
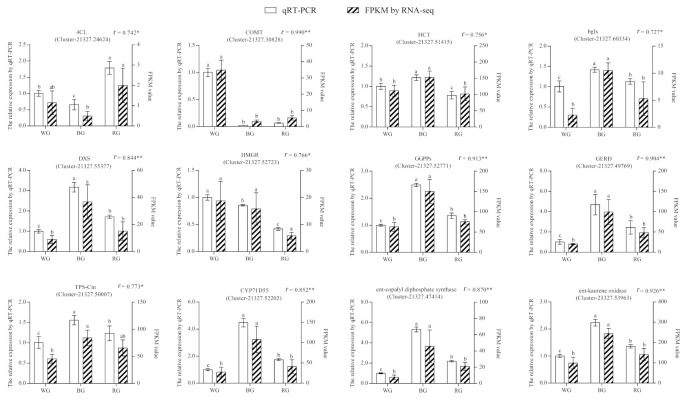
qRT-PCR validation of 12 selected unigenes in WG, RG, and BG from *S. glabra*. The left *Y*-axis and white legend represent the relative expression levels as determined by RT-qPCR, while the right-axis and slash legend show the FPKM values. Expression values were adjusted by setting the expression of WG to 1 for each unigene. All RT-qPCRs for unigenes were accomplished in triplicates, with two repeats per experiment. Error bars indicate SD, and different lowercase letters (a–c) represent significant differences among the three samples at *p* < 0.05. The correlation coefficient (R^2^) between RT-qPCR and RNA-seq for each unigene is shown with its corresponding significance level (* represents *p* < 0.05, ** represents *p* < 0.01).

To validate the RNA-seq results, we selected 12 DEGs (phenylpropanoid and terpenoids biosynthetic genes) and analyzed their expression levels in WG, BG, and RG using qRT-PCR (Figure 7 and Supplementary Table S10). The expression patterns of these genes were consistent with RNA-seq results, with correlation coefficients (R^2^) > 0.7. The results showed that RNA-seq data were accurate and can be used in future experiments.

## 3. Discussion

Plant root systems play a crucial role in absorbing water and nutrients, promoting propagation and phytochemicals synthesis, and decomposition. In addition, roots adapt to the changing environments and interact with other organisms, such as mycorrhizae and pathogens [27,29]. To date, considerable emphasis has been given to understanding the different factors that influence root growth and development. Numerous investigations have revealed that the root development network is a complex process [27]. Among these factors, the combined activity of endogenous phytohormones, such as auxins, cytokinin, brassinosteroids (BRs), and strigolactone among others are recognized as the main regulators [27,30,35,36,37,38,39]. Therefore, in this study, we performed root morphological analysis and carried out transcriptomic and metabolomic profiling to elucidate the effects of different LED lights on the root tissues of *S. glabra*.

### 3.1. Root Morphological Analysis

We found that under the BL cultivation, it was conducive to increment in root fresh weight, root-shoot ratio, root length, root surface area, and root volume. At the same time, the values of these indexes decreased significantly in RG, compared to WG. In KEGG enrichment analysis, we found that some DEGs were enriched in BRs biosynthesis and plant hormone signal transduction. In the BRs biosynthetic pathway, the expression level of the unigene (Cluster-21327.57603) encoding BR6ox1/2 (brassinosteroid-6-oxidase 1/2, belonging to CYP85A family) was down-regulated, but the unigene (Cluster-21327.54243) encoding BAS1 (PHYB activation tagged suppressor 1) was highly expressed in BG. In contrast, the unigene (Cluster-21327.58643) encoding DWF4 (steroid 22-alpha-hydroxylase, CYP90B1) was significantly down-regulated in RG. BRs are steroid hormones critical for plant growth and development, including the growth of the root system [29,40,41,42,43]. BR6ox1/2 and BAS1 are the rate-limiting downstream enzymes in BRs biosynthesis [44]. BR6ox1/2 are involved in the conversion of 6-Deoxocastasterone to castasterone and brassinolide [45]. Previous studies have revealed that *br6ox1/2* mutants were phenotypically different from the wild type, e.g., smaller leaves, shorter hypocotyls, and thicker stems [44,45,46]. BAS1, belonging to the cytochrome P450 monooxygenase superfamily, inactivates BRs and modulates cotyledon expansion, repression of hypocotyl elongation, and flowering time [47,48]. Some studies showed that the supply of BRs at lower concentrations promoted root elongation, whereas higher concentrations of BRs or some BR-deficient mutants suppressed root growth [29,43]. In our study, the different expression patterns of *BR6ox1/2* and *BAS1* genes increased the production of compounds 26-Hydroxycastasterone and 26-Hydroxybrassinolide, and reduced the yield of BRs, such as castasterone and brassinolide. Our study results, therefore, suggested that lower concentrations of endogenous BRs might promote root growth, due to lower production of BRs. The *DWF4* gene mediated multiple 22α-Hydroxylation steps in BR biosynthesis, and *dwf4* mutants showed a dwarf phenotype, including the short-roots phenotype [49,50]. Under our experimental conditions, the RL inhibited root growth, which could be related to the down-regulation of the *DWF4* gene. In addition, some DEGs were also mapped in the signal transduction pathway of other plant hormones. In the group of BG vs. WG, the unigene (Cluster-21327.50210), encoding DELLA repressor proteins were significantly up-regulated in BG. The DELLA proteins are key gibberellin (GAs) signaling components, which are involved in auxin and ethylene signaling pathways and play a vital role in plant development, and response to abiotic and biotic stresses [32,51]. DELLA proteins are degraded by GAs and their accumulation is highly dependent on the concentration of GAs [31,32,52]. Under adverse conditions, *Arabidopsis* could survive via reduction of GA level and rapidly increase in DELLA proteins [51]. Under BL irradiation, the seedlings of *S. glabra* had shorter hypocotyls and a smaller leaf area [16]. Moreover, malondialdehyde (MDA) content, peroxidase (POD) activity, and superoxide dismutase (SOD) activity were much higher in leaves (data not shown). In response to the abiotic stress, the enhancement of POD and SOD activity could protect seedlings from oxidative damage by ROS (reactive oxygen species), and the accumulation of DELLA proteins might be beneficial to the survival of seedlings. The unigene (Cluster-21327.46194), encoding ARF (auxin response factor) transcription factor (TF), had a higher expression level in BG, relative to WG. The ARF TFs specifically bind AuxRE (auxin response cis-acting element) to regulate many auxin-related genes associated with developmental processes, such as cell elongation and division, hypocotyl elongation, and root growth [53,54]. The increased expression of the *ARF* gene might promote root growth of *S. glabra* under BL conditions.

### 3.2. Phenylpropanoid Metabolism Biosynthesis

In phenylpropanoid biosynthesis, we put more emphasis on the accumulation patterns of phenolic acids, lignans and coumarins, and flavonoids compounds in roots. For phenolic acids, the production of sinapyl alcohol and sinapic acid significantly decreased in BG and RG, whereas chlorogenic acid substantially increased in BG. According to the transcriptome profiling results (Figure 6A,B), the expression levels of upstream genes, including *COMT* (Cluster-21327.30828), *F5H* (Cluster-21327.143), and *CAD* (Cluster-21327.61286), were significantly down-regulated in BG and RG, which might lead to a reduction in contents of sinapyl alcohol and sinapic acid. Chlorogenic acid is one of the naturally occurring compounds with abundant phenolic compounds having antioxidant properties. Notably, chlorogenic acid is formed by several continuous enzymatic steps, from the intermediate of 4-coumaric acid [55,56]. HCT, which plays a vital role in the biosynthesis of chlorogenic acid, is encoded by Cluster-21327.51415. The chlorogenic acid content was positively correlated with higher expression levels of *HCT* gene in BG.

Coumarins are widely distributed in numerous plant species and are involved in defense against phytopathogens, abiotic stress, oxidative stress, and clinical diseases. Furthermore, coumarins are the primary secondary metabolites of *S. glabra* [57,58,59]. Even though it is well known that coumarins are produced via the phenylpropanoid pathway, the specific biosynthetic pathways of most coumarins are obscure [57,60]. Here, we selected several pivotal coumarins and compared the compositional differences among samples (Figure 3). Among these compounds, the contents of fraxetin and 6-methylcoumarin significantly decreased in BG and RG. Moreover, the production of isofraxidin, scopoletin, fraxidin, and 3, 4-dihydrocoumarin slightly reduced. During coumarine biosynthesis, bglx (EC3.2.1.21), belonging to GH1 (glycosyl hydrolase family 1) β-glucosidases, catalyses the conversion of β-d-glucosyl-2-coumarinate to coumarinate, which then transforms into coumarine [61,62,63]. We identified two DEGs encoding bglx enzymes; the unigene (Cluster-21327.60334) had the highest expression level in BG, whereas Cluster-21327.49482 was significantly down-regulated in both BG and RG. In this study, the compound of coumarine was not identified, but coumarine derivatives were found in root tissue, such as 6-methylcoumarin and 3,4-dihydrocoumarin. The contents of them were reduced to various degrees in BG and RG under BL and RL conditions. Unfortunately, the following catalyzing steps related to 6-methylcoumarin and 3,4-dihydrocoumarin are still not unveiled. Therefore, we could not identify a reduction in contents of two coumarine derivatives caused by specialized enzymes.

This study identified a crucial enzymatic step related to the biosynthesis of scopoletin, scopolin, and fraxetin, which are derived from the intermediate compound ferulic acid and then catalyzed by 4CL, F6’H1, SGTF, and S8H (Figure 6). Both F6’H1 and S8H are members of the 2-oxoglutarate (2OG) and Fe(II)-dependent oxygenase superfamily proteins. F6’H1 is a crucial enzyme that exhibits orthohydroxylase activity for feruloyl CoA to form 6’-hydroxyferuloyl CoA, followed by trans/cis isomerisation of the side chain and lactonisation to form scopoletin [64]. After that, S8H converts scopoletin to fraxetin, while SGTF catalyses transformation from scopoletin to scopolin [64,65]. In this research, we found that the DEGs of Cluster-21327.57168 and Cluster-21327.47660, encoding F6’H1 and SGTF, respectively, were down-regulated in RG, and the production of the corresponding compounds also reduced. Apart from scopoletin formation from feruloyl CoA by F6’H1, some studies showed that scopoletin could also result from the conversion of esculetin, via methylation of O-MT [57]. The unigene (Cluster-21327.49256), encoding O-MT had a lower expression level in RG, which could explain the reduction in the yield of scopoletin.

### 3.3. Terpenoids Metabolism and Biosynthesis

For terpenoids, we identified nine triterpenoids and one sesquiterpenoid (prehelminthosporol) in root tissues of *S. glabra* using UPLC-ESI-MS/MS. However, we did not identify monoterpenoid compositions in this study because GC/MS was not performed. Moreover, relative to WG, the production of eight terpenoids substantially reduced in BG. On the other hand, the contents of only two terpenoids showed considerable change in RG.

During terpenoid backbone biosynthesis, plant isoprenoids (IPP and DMAPP) are generated via the MVA pathway in the cytosol or the MEP pathway in plastids, while HMGR and DXS are the rate-limiting enzymes in both pathways [66,67]. It has been extensively shown that HMGR has several isoforms in higher plants, and the expression patterns of the various isoforms vary in different tissues [68,69,70]. Likewise, the DXS enzyme is encoded by a multigene family, and the various genes exhibit differential expressions at different growth stages or tissues [71,72]. For HMGR enzyme, we identified two DEGs encoding it, and both of them had the lowest expression levels in RG. The gain or loss of function mutations in *DXS* genes either impaired or enhanced the contents of isoprenoids [72,73]. For DXS enzyme, Cluster-21327.52062 and Cluster-21327.55377 encoded it, and their expression levels were significantly up-regulated in BG, whereas the expression of Cluster-21327.54217 had a significant decrease in RG. In the subsequent biosynthetic pathways for various classes of terpenoids, some committed enzymes had different expression patterns. In monoterpenoids, for instance, the DEGs encoding TPS-cin (Cluster-21327.50007) and α-terpineol synthase (Cluster-21327.53297) had higher expression levels in BG. Moreover, 1, 8-cineole, a natural oil compound, is the direct product of these successive enzymatic reactions, and up-regulation of these DEGs could enhance the yield of this compound. For sesquiterpenoid biosynthesis, the expression of the downstream genes of Cluster-21327.49769 encoding GERD and Cluster-21327.52202 encoding CYP71D55 were also significantly up-regulated in BG, which could increase the production of (-)-germacrene D, solavetivol, and solavetivone. In addition, the DEGs, associated with the biosynthesis of GAs, had a higher expression level in BG, which could enhance the accumulation of down-stream secondary metabolites. As described above, the results indicated that BL up-regulated the expression levels of some crucial genes in terpenoids biosynthesis, which was likely to accumulate much more production of corresponding terpenoids. Due to the complexity of terpenoid biosynthesis and regulation, the mechanism by which the production of the identified terpenoids in BG was reduced remained obscure.

## 4. Materials and Methods

### 4.1. LED Devices

The spectral distributions of WL (peak at 380 ~ 760 nm), RL (peak at 656 nm), and BL (peak at 450 nm) were measured using an HR-450 machine. The photosynthetic photon flux density (PPFD) of each treatment was 80 μmol·m^−2^·s^−1^, while the photoperiod was 16/8 h (day/night). PPFD was also measured using HR-450 machine. The LED lights and light meter (HR-450) were procured from Hipoint Corporation (Hipoint Co., Gaoxiong, China).

### 4.2. Plant Materials

The collected seeds of *S. glabra* were planted in seedling-raising disks (30 × 20 × 10 cm) containing Pindstrup seeding growth media (Pindstrup Co., Helsingor, Denmark), at constant temperature and humidity incubator (25 °C and 60% humidity), at the Herb Institute of the Fujian Agriculture and Forestry University, Fuzhou, Fujian Province, China (26°5′ N, 119°13′ E). When the first leaves were fully expanded, the 90 seedlings were transplanted into black PVC rectangular pots (10 × 10 × 8 cm) containing Pindstrup substrate media (NO.5, pH 5.5). All the seedlings were randomly divided into three groups and placed under three different light treatments (WL, BL, and RL) for 60 days in the culture room (25 °C and 30% humidity). In this study, we primarily focused on the effects of WL, RL, and BL on the root tissues of *S. glabra*. After that, the roots of 9 healthy seedlings were separated from seedling, and triplicate composite samples were made from each treatment. Finally, nine samples (WG1/2/3, BG1/2/3, and RG1/2/3) were immediately frozen in liquid nitrogen and stored at −80 °C until further analysis.

### 4.3. Root Morphological Analyses

After a 60-day growth, 14 seedlings from each treatment were uprooted, carefully washed, detached, directly placed on the scanner glass, and digitized using a root scanner (professional model). The root morphological parameters, including total length, surface area, and total volume were then evaluated by the WinRHIZO^TM^ software [74,75]. Furthermore, the software also assigned different indexes to predefined diameter classes, thus providing diameter distributions of various root morphology traits.

### 4.4. RNA Extraction and Transcriptome Profiling

Total RNA extraction was performed by the RNAprep Pure Plant Kit (DP441, TIANGEN BIOTECH Co., Beijing, China), according to the protocol of the manufacturer. The total RNA samples were then treated with DNase I (Takara Co., Kusatsu, Shiga, Japan) to eliminate genomic DNA contamination and sent to the Novogene Bioinformatics Technology Company (Beijing, China) for cDNA library construction and Illumina sequencing. 

### 4.5. De Novo Assembly and Annotation

For high quality assembly, clean reads were obtained by removing adaptor sequences and reads with more than 10% unknown bases. Low-quality reads, in which the percentage of quality value (≤20) base exceeded 50%, were also deleted. The Trinity method was employed for *de novo* assembly of clean reads [76]. Three public databases/programs were used to annotate the unigenes, including the NCBI non-redundant protein (Nr), the NCBI nucleotide sequences (Nt), and the Kyoto Encyclopedia of Genes and Genomes (KEGG).

### 4.6. Gene Expression Analysis

FPKM (fragments per kilobase of transcript per million fragments) values were used to analyze gene expression [77]. Differential expression analysis of any two treatment sets was accomplished by the R package DEGseq2 [78]. An adjusted *p*-value of 0.05 was set as the threshold to determine significant differences in differentially expressed genes. The KEGG pathway was annotated using the KEGG database. The corrected *p*-value < 0.05 and |log_2_FoldChange| > 1 were set as the threshold to evaluate for significant differences in the KEGG enrichment analyses. The heat map diagram was constructed using the R package pheatmap. To visualize the similarities in gene expression between samples, correlation analysis (Pearson’s) was performed by the R package cor function.

### 4.7. qRT-PCR Confirmation of the Gene Expression Profile

First-strand cDNA was generated from about 1 μg of total RNA using PrimeScriptTM RT reagent Kit with gDNA Eraser (RR047A, Takara Co., Japan), according to the manufacturer’s instructions. Gene-specific primers for qRT-PCR were designed using the Primer Premier 5.0 software (Primer, San Francisco, CA, USA) and synthesized by the SunYa Biotech (Fuzhou, China) company. *CAC* (clathrin adaptor complexes) was selected as the reference gene. All primers used are listed in Supplementary Table S11. The qRT-PCR was performed on the ABI 7500 Real-Time PCR system (Applied Biosystems, Carlsbad, CA, USA), using SYBR Green premix Ex Taq Kit (RR820A, Takara Co., Japan). Each reaction mixture was 20 μL, including 1 μL of diluted first-strand cDNAs (100 ng·μL^−1^), 0.8 μL of each primer (10 μmol·L^−1^), 10 μL of SYBR Green Premix Ex Taq, 0.4 μL of RoxII and 7 μL of ddH_2_O. The amplification program of qPCR was run as follows: 95 °C for 30 s, followed by 40 cycles of 95 °C for 5 s and 60 °C for 34 s and associated with each primer specific annealing temperature 95 °C for 15 s, 60 °C for 1 min and 95 °C for 15 s in 96-well optical reaction plates. Expression levels of the tested reference genes were determined by CT values and calculated by 2^−ΔΔCT^ method. All analyses were repeated two times in each biological replicates.

### 4.8. Metabolite Profiling Analysis

#### 4.8.1. Sample Preparation and Extraction

The nine freeze-dried root samples (WG1/2/3, RG1/2/3, BG1/2/3) were placed in vacuum freeze-drying equipment (Scientz-100F, Scientz Co., Ningbo, China), and then crushed using a mixer mill (MM 400, Retsch, Hamburg, Germany) with a zirconia bead for 1.5 min at 30 Hz. Root powder (100 mg) was incubated in 0.6 mL of 70% aqueous methanol overnight at 4 °C Following 10,000× *g* for 10 min, the extracts were absorbed and filtrated before UPLC-MS/MS analysis (UPLC, Shim-pack UFLC SHIMADZU CBM30A system, Japan; MS, Applied Biosystems 4500Q TRAP, USA). The UPLC and ESI-Q TRAP-MS/MS conditions were displayed in the previous study [16,79].

#### 4.8.2. Metabolite Identification and Analysis

Metabolite identification was conducted by the self-compiled MWDB database (MetWare biological science and Technology Co., Ltd., Wuhan, China) and publicly available metabolite databases. Quantitative analysis of metabolites was accomplished based on the MRM mode, and the characteristic ions of each metabolite were screened through the QQQ mass spectrometer to obtain signal strengths. Integration and correction of chromatographic peaks were performed using MultiQuant version 3.0.2 (AB SCIEX, Framingham, MA, USA). The corresponding relative metabolite contents were shown as chromatographic peak area integrals. Then, correlation analysis was performed to observe the reliability of metabolic composition within nine root samples. K-mean clustering analysis was applied to analyze the accumulation patterns of compounds and visualize the similarities and differences between WG, BG, and RG. The relevant metabolite contents were treated with centroid initialization and standardized processing, after which we performed K-mean analysis. Metabolites with significantly different contents were set with thresholds of variable importance in projection (VIP) ≥ 1 and fold change ≥2 or ≤0.5.

### 4.9. Statistical Analysis

SPSS 20.0 software (IBM Corp., Armonk, NY, USA) was used for statistical analysis. Differences in comparison of mean between different treatments were performed by analysis of variance (ANOVA) at α 0.05, 0.01, 0.001, and 0.0001 probability level. Where * represents *p* < 0.05, ** represents *p* < 0.01, *** represents *p* < 0.001, **** represents *p* < 0.0001. All the values were presented as mean ± standard errors. Graph-Pad Prism 8.0 (GraphPad, San Diego, CA, USA) was used for graphs.

## 5. Conclusions

Under WL, RL, and BL environments, the root fresh weight and morphological traits of *S. glabra* seedlings exhibited significant differences. The results showed that BL promoted root growth, while RL inhibited it. The transcriptomic and metabolomic analysis demonstrated that (1) In BR biosynthesis, the coactions of *BR6ox1/2* and *BAS1* genes probably promoted root development under BL treatment, whereas down-regulation of the *DWF4* gene could inhibit root growth in the RL treatment. Furthermore, genes related to plant hormone signal transduction also affected BR biosynthesis. (2) We selected 32 principle active metabolites from the identified 433 compounds. The contents of sinapyl alcohol, sinapic acid, fraxetin, and 6-methylcoumarin decreased significantly in BG and RG. In contrast, a substantial increase was observed in chlorogenic acid, rosmarinyl glucoside, quercitrin, quercetin, and phloretin in BG. Moreover, the production of eight terpenoids was much lower in BG. Higher or lower concentrations of specific secondary metabolites were possibly caused by the up or down-regulated expression levels of key genes, e.g., *COMT*, *F5H*, *HCT*, *OMT*, *SGTF*, *DXS,* and *HMGR*. Collectively, in the present study, it appeared that BL was advantageous having various positive effects on growth, development, and accumulation of some bioactive metabolites in root tissues of *S. glabra*. In future studies, appropriately increasing the proportion of light source (BL) may be suitable for rapid and large-scale management of *S. glabra* production.

## Data Availability

9 transcriptome raw data produced by Illumina NovaSeq 6000 have been deposited in the Genome Sequence Archive (SRA) database (https://bigd.big.ac.cn/gsa/, accessed on 30 June 2021) under the accession number CRA002640.

## Supplementary Materials

The following are available online at https://www.mdpi.com/article/10.3390/ijms22137179/s1, Table S1: The statistics of root length, root surface area, root volume, and the number of root tips under WL, RL, and BL; Table S2: The statistics of the detected metabolites in root tissues of *S.glabra*; Table S3: K-means clustering analysis of all the detected metabolites; Table S4: The differentially produced metabolites between WG and BG; Table S5: The differentially produced metabolites between WG and RG; Table S6: The differentially produced metabolites between RG and BG; Table S7: The relative content of the 32 principle active metabolites; Table S8: The DEGs in groups of WG vs. BG, WG vs. RG and RG vs. BG; Table S9: The FPKM values of some identified unigenes; Table S10: The qRT-PCR data of 12 selected unigenes; Table S11: Gene-specific primers for RT-qPCR.
