# Supplementary material for: Consequences of LED Lights on Root Morphological Traits and Compounds Accumulation in Sarcandra glabra Seedlings
Source: Int J Mol Sci. 2021 Jul 2;22(13):7179. doi: 10.3390/ijms22137179 (PMC8268991; doi:10.3390/ijms22137179)
Supplement: Supplementary file 1 [file ijms-22-07179-s001.zip › Supplementary11.pdf]

Table S1 Gene-specific primers for RT-qPCR

Name of gene	Foward /Reverse Primer	product size (bp)
4CL	F:5’ATTACACACTTGCCGCTTGC3’<br>R:5’AAACACCCAAAGACGGCATC3’	86
COMT	F:5’TCAGTTCGCCATGCAATTGG3’<br>R:5’TGATCTCCAACACTTCGAGCTC3’	83
HCT	F:5’ACAATGCAGATGCCACTGTG3’<br>R:5’TGTGCATGCAACAACGATGC3’	107
bglx	F:5’GCTCGAAATTCGTCAAGTCCAG3’<br>R:5’TGTCAGGTTCATGCTGTCAC3’	98
DXS	F:5’ATGTAGCGTACATGGCATGC3’<br>R:5’ACCATGTGCATTAGCTCAGC3’	76
HMGR	F:5’TTTCAGGTGGGTACTGTTGGG3’<br>R:5’TTGCACCTTTCACACCAAGC3’	82
GGPPs	F:5’ATTGGGCCTCCATAAATGCC3’<br>R:5’TCAAATGGTGCATGGGATCG3’	96
GERD	F:5’AGCAACCAGGGAAGCTTTTG3’<br>R:5’TGGAAGTAGCAGCATGTCCAG3’	134
TPS-cin	F:5’TGTTCAGCCATGGTTCTTCG3’<br>R:5’ACTCCGCGACTGAATGAAAC3’	93
CYP71D55	F:5’TAGCGAATGTGAAGCTTCCG3’<br>R:5’TGGCAAACAAGCACAGGTTG3’	144
ent-copalyl diphosphate synthase	F:5’TCGTACTTGGAAGGGTGCAC3’<br>R:5’TCCTTTCCAGCCTGATCTGTTG3’	87
ent-kaurene oxidase	F:5’TTCTCAACCATGGCTTTCGC3’<br>R:5’AGTGCCTTCTTCGAGTGATGAG3’	88
CAC	F:5’TCCGACAAATTGGAGGTTGC3’<br>R:5’TGCTGCTGACAACAATCACG3’	75
